# Risk factors for postage stamp fracture following arthroscopic Bankart repair: a systematic review

**DOI:** 10.1016/j.jseint.2025.09.017

**Published:** 2025-10-24

**Authors:** Jakob Oury, Srikanth Mudiganty, Alexander Crowley, Brian Godshaw

**Affiliations:** a University of Queensland-Ochsner Clinical School, New Orleans, LA, USA; b Ochsner Andrews Sports Medicine Institute, Jefferson, LA, USA

**Keywords:** Shoulder dislocation, Arthroscopy, Bankart lesion, Suture anchors, Fractures, Risk factors

## Abstract

**Background:**

Postage stamp fractures are a rare but considerable complication after arthroscopic Bankart repair. Previous literature has implicated various patient- and technique-specific factors, but analyses often lacked comparison to the broader operative sample. The purpose of this study is to systematically review the literature on postage stamp fracture after arthroscopic Bankart repair, evaluate patient- and anchor-specific risk factors, and contextualize findings within the full operative sample.

**Methods:**

A systematic review was conducted to identify studies reporting postage stamp fractures following arthroscopic Bankart repair using Preferred Reporting Items for Systematic Reviews and Meta-Analyses guidelines. Patient demographics, anchor characteristics, and surgical outcomes were extracted. Risk factor associations were evaluated descriptively, with emphasis on whether reported associations held true across the full sample of surgical cases in each study.

**Results:**

A systematic search yielded 2,349 articles; after duplicate removal and screening, 7 studies met inclusion criteria (2 comparative, 5 noncomparative). Studies included a total of 1,264 patients aged 10-61, with fracture incidence in larger cohorts ranging from 1.58% to 5%. Most patients with fractures participated in sports, particularly contact or high-risk activities. Although male patients made up the majority of fracture cases, this reflected the underlying demographic of operative patients. Patients with fractures were often the same age or older than the overall sample. Anchor number, diameter, type, and material were variably reported. Average anchor count was not consistently higher in patients with fractures, and no study found a significant association between anchor quantity and fracture risk. Absorbable anchors were implicated in a higher proportion of fracture cases. Osteolysis was common among patients with fractures. Insertion angle and suture type (knotted vs. knotless) were inconsistently reported and not clearly linked to fracture incidence.

**Conclusion:**

Male sex and younger age, previously identified as risk factors, were not associated with postage stamp fractures when contextualized within the full operative sample. Presence of osteolysis and athletic participation was consistently associated with postage stamp fracture and may represent meaningful risk factors. Contrary to earlier reports, higher anchor quantity was not associated with increased postage stamp fracture risk; instead, lower anchor counts may be linked to instability, which itself may predispose to postage stamp fracture. Use of absorbable anchors emerged as a potential contributor to fracture risk that warrants further research.

The labrum of the shoulder plays a crucial role in maintaining glenohumeral stability by deepening the glenoid fossa and serving as an attachment site for ligaments and the long head of the biceps tendon.^11^ Labral injuries such as Bankart lesions can be common in athletes and individuals experiencing recurrent shoulder dislocation.^17^ Arthroscopic labral repair using suture anchors is the standard of treatment to restore stability and function.^2^ Suture anchors, typically composed of metallic, bioabsorbable, or hybrid materials, are drilled into the glenoid rim and allow for secure reattachment of the labrum. A rare but notable complication of shoulder labrum repair using suture anchors is fracture of the glenoid rim, sometimes referred to as a “postage stamp” fracture (PSF). It is thought that drilling suture anchors compromises the structural integrity of the glenoid rim, leading to this fracture pattern.^9^ There are a number of factors that potentially increase the risk of glenoid rim fracture, which can be divided into anchor-specific and patient-specific factors.

At the moment, there is no consensus for the optimal number of suture anchors to use. Some studies recommend using at least 3 anchors to ensure joint stability, saying fewer anchors put the patient at risk of repair failure or recurrence.^16^^,^^18^ This is a debated point, as a 2017 systematic review refutes this claim, stating there is no difference in repair stability when using fewer anchors.^4^ While biomechanical studies have shown that increasing the quantity of suture anchors used in repair can increase the risk of PSF, this relationship has yet to be defined in-vivo.^9^ A 2019 systematic review identified the presence of more than 3 suture anchors as a risk factor for fracture; however, a 2023 systematic review analyzing 245 Bankart repairs found that the mean quantity of anchors used ranged from 3.92 to 7.1.^7^^,^^19^ Rather than implicating suture anchor quantity as an independent risk factor, these findings may instead suggest that arthroscopic Bankart repair itself predisposes patients to fractures at the anchor sites, irrespective of anchor quantity. Additional anchor-based factors that may influence fracture risk include anchor type (knotted vs. knot-less), material, and insertion angle.^14^^,^^19^

Patient-specific factors that may contribute to PSFs include sex, age, and level of activity.^19^ While a 2019 systematic review on PSF reported higher incidence in men aged 25 years and younger, these conclusions warrant careful interpretation.^19^ The predominance of male patients in most of their reviewed studies evaluating Bankart repair introduces a selection bias, making it difficult to determine whether sex is an independent risk factor or merely a reflection cohorts studied.^10^ In 2 large cohort studies evaluating postoperative recovery in Bankart repair patients, the male-to-female ratios were 570:0 and 214:43.^12^^,^^15^

Similarly, the designation of age as a risk factor is confounded by activity level. Younger patients are more likely to engage in sports and physical activity, which may explain the association with glenoid rim fractures.^5^ As prior studies have already identified sporting activity as a key contributor to fracture risk, it remains unclear whether younger age itself is an independent risk factor or simply a surrogate marker for increased physical demands placed on the repaired labrum.^19^

While previous studies have identified both anchor-specific and patient-specific factors as potential contributors to PSF, closer examination reveals the need for further investigation. This systematic review is aimed to clarify true risk factors, guide surgical decision-making, and contribute to the growing body of arthroscopic research, ultimately improving patient outcomes. Authors hypothesize that risk factors for PSF for patients after arthroscopic Bankart repair will include increased density or number of anchors, large-diameter anchors, as well as inappropriate angle on anchor insertion in addition to patient factors like poor bone quality.

## Materials and methods

### Eligibility criteria

This study aimed to find, assess, and synthesize randomized control trials, cohort studies, case-control studies, and case series studies. The Population, Intervention, Comparison, and Outcome framework was used to define the research question and guide development of inclusion and exclusion criteria. The following inclusion criteria were set: all levels of evidence above case report in peer-reviewed journals, studies published in English with viewable abstract, human patients of all ages receiving arthroscopic Bankart repair, studies reporting incidence of PSF after intervention, and studies reporting patient and suture anchor details. The following exclusion criteria were used: studies focusing on anchor use in other joints (ie, hip), case reports, studies focusing on nonsurgical repair techniques, biomechanical studies, studies not reporting PSF incidence or reporting incidence without prior Bankart repair with suture anchors.

## Search strategy

Four online databases (Pubmed, Embase, Scopus, and Web of Science) were searched for literature relevant to arthroscopic repair of shoulder labrum tears. Preferred Reporting Guidelines for Systematic Reviews and Meta-analyses guidelines were followed. The search was conducted on March 27, 2025, including all articles from January 1, 2000, to search date. Keyword-derived Boolean logic search strings used on each database is shown below:

Pubmed/Embase/Scopus: ((suture anchor) OR (anchor density)) AND ((labrum repair) OR (Bankart repair) OR (arthroscopic Bankart) OR (shoulder stabilization)) AND ((fracture) OR (glenoid rim)).

Web of Science: term search (TS) = ("suture anchor" OR "anchor density") AND TS = ("labrum repair" OR "Bankart repair" OR "arthroscopic Bankart" OR "shoulder stabilization") AND TS = ("fracture" OR "glenoid rim"). Filters and limits included the aforementioned search dates and English language. The search criteria and method were replicated on April 13, 2025.

### Study screening

**Figure 1:**
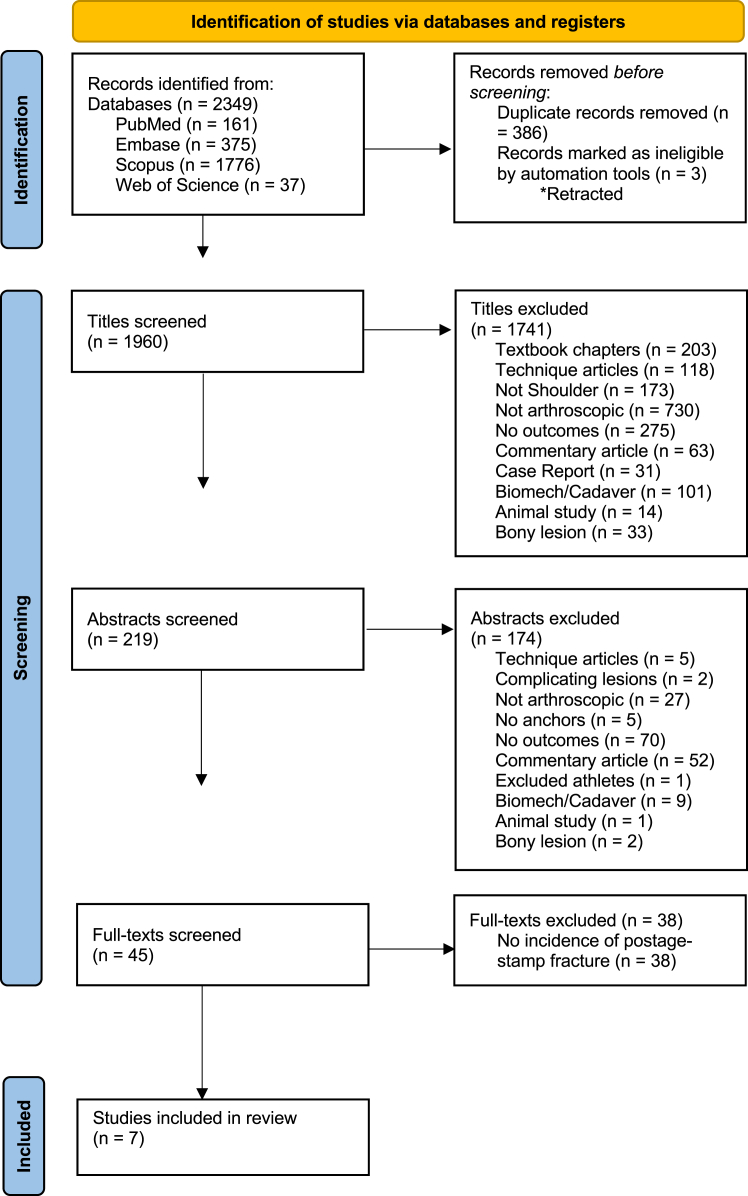
PRISMA flow diagram depicting article screening process. *PRISMA*, Preferred Reporting Items for Systematic Reviews and Meta-Analyses.

Screening by title and abstract was conducted by 2 authors; both retrieved full-text for screening and inclusion. Citations of relevant systematic reviews were screened by one author. Discrepancies were resolved by consensus or by referring to a third author. Figure 1 shows the Preferred Reporting Items for Systematic Reviews and Meta-Analyses flow diagram for the selection process, including reasons for exclusion.

## Data extraction

Study characteristics and outcomes from each study were recorded in a Microsoft Excel spreadsheet. Extracted information included author and year of publication, level of evidence, study design, sample size, incidence of PSF, full-sample patient demographics, demographics of reported fractures, full-sample anchor details, anchor details of reported fractures, full-sample presence of osteolysis and presence of osteolysis in reported fractures, and study specific relevant findings. Patient demographics included gender, age, time to fracture, participation in athletics, and participation in collision or high risk athletics. Anchor details included quantity, diameter, type (knotted or knot-less), material, and insertion angle.

### Assessment of quality

Included studies were assessed using the methodological index for nonrandomized studies (MINORS) criteria as to maintain continuity with the systematic review from 2019. Each study was given a score of 0, 1, or 2 for 8- 12 criteria depending on whether the study was comparative or noncomparative. MINORS criteria can be visualized in Table I.

**Table I:** MINORS criteria of included studies.

Criterion	Park et al^14^	Nakagawa et al^12^	Banerjee et al^3^	Narbona et al^13^	Fritsch et al^6^	Augusti et al^1^	Park et al^15^
Clearly stated aim	2	2	1	2	1	2	2
Inclusion of consecutive patients	1	2	0	1	0	2	2
Prospective collection of data	0	2	0	0	1	2	2
Appropriate endpoint	2	2	2	2	2	2	2
Unbiased assessment of study endpoint	1	2	0	1	0	2	2
Appropriate follow-up	1	2	2	2	1	1	2
Lost to follow-up < 5%	0	2	0	1	0	0	2
Prospective calculation of study size	0	0	0	0	0	0	0
Adequate control	2	2	NA	NA	NA	NA	NA
Contemporary groups	2	2	NA	NA	NA	NA	NA
Baseline equivalence	1	1	NA	NA	NA	NA	NA
Adequate stat analysis	2	1	NA	NA	NA	NA	NA
Total	14	20	5	9	5	11	14

*MINORS*, methodical index for nonrandomized studies; *NA*, not applicable.

### Statistical analysis

At each stage of screening, a kappa value was calculated to determine inter-reviewer agreement. Kappa values were calculated using GraphPad interrater agreement calculator. Kappa values are interpreted using Landis and Koch's interpretation guide.^8^

Due to low level of evidence, meta-analysis was not performed. Instead, a descriptive synthesis was performed of the included studies. Given the rarity of glenoid rim fractures following anchor-based repair and the limited available data, a structured descriptive synthesis offers value to identify potential trends and recurring associations. Variables from the comprehensive data extraction table were descriptively compared across studies. Where relevant, crude proportions or average values were reported to highlight consistent patterns. This approach allows for cautious pattern recognition without overstating precision or causal inference. The limitations of this synthesis approach, including risks of bias and confounding, are further addressed in the Discussion section.

## Results

### Study characteristics and reviewer agreement

The search of Boolean logic yielded 2,349 total articles across 4 databases, with 1,960 titles to review after filtering duplicates and retracted studies. Seven studies met inclusion criteria: 2 cohort studies and 5 case series. Interrater agreement was substantial at the title review stage (κ = 0.696; 95% confidence interval 0.667-0.724), almost perfect at the abstract review stage (κ = 0.842; 95% confidence interval 0.749-0.974), and perfect at full-text review (κ = 1.0). Two studies were comparative, the remaining 5 were noncomparative. The average MINORS score for comparative studies was 17.0 out of 24 and the average for noncomparative studies was 8.8 out of 16.

#### Patient demographics

A total of 1,264 patients were included across the 7 studies aged 10-61. Three studies employed a total patient sample of 12 or fewer patients.^1^^,^^3^^,^^6^ Furthermore, 3 studies included more than 250 total patients with reported incidence of PSF of 5%, 2.1% and 1.58%.^12^^,^^13^^,^^15^ Patient gender was reported in 5 studies with 3 studies using 100% male samples, the other 2 using 97.4% and 83.3% male samples.^1^^,^^3^^,^^6^^,^^12^^,^^14^ Two studies reported fracture in female patients.^12^^,^^14^ One of those studies had 1 of 1 female patients report PSF.^14^ In the other study, 2 of 43 (4.65%) female patients suffered PSF.^12^ In that same study, 5.14% of male patients suffered PSF (11 of 214).^12^ In both studies further analysis revealed PSF likelihood was not significantly different based on gender. Total average age was reported in 4 studies, 3 of which had a mean total age of less than 24 years.^1^^,^^3^^,^^6^^,^^13^ Average age in fracture patients was the same or greater in all 4 of those studies. All 7 papers reported average age in fracture patients with 4 studies reporting an average age of less than 24. Average age in fracture patients ranged from 16.7 years to 32.7 years (Table II). The mean interval between initial Bankart repair and PSF occurrence was greater than 24 months in 3 of 7 studies (n = 21) and less than 24 months in the remaining 4 studies (n = 44). Of the 7 studies, 5 reported total sample's participation in sports and all 7 studies mentioned sports participation in fracture patients (Table II). In all 7 studies, athletes made up more than 63% of the patients that suffered a PSF. There are 2 studies which reported that participation in collision (ie, rugby, American football), high-risk (ie skiing, autoracing), or overhead sports (ie, weightlifting, volleyball) played a role in fracture incidence.^12^^,^^14^ There was one incidence of low-energy mechanism causing fracture (pulling a door handle); however, this was complicated by a motor vehicle accident 4 weeks earlier.^6^ A summary of patient demographic data is shown in Table II.

**Table II:** Patient characteristics.

Author	Year	Level	Sample size	PSF incidence	M/F (total)	M/F (fracture)	Mean age (total)	Age range (total)	Mean age (fracture)	Age range (fracture)	Mean mo to fracture	Sport			
												Total	Collision/high-risk (total)	Fracture	Collision/high-risk (fracture)
Park et al^14^	2020	II	39	19	38/1	18/1	NR	18-49	24.9	18-49	8	16	NR	12	NR
Nakagawa et al^12^	2017	III	257	13	214/43	11/2	NR	10-40+	16.7	14-19	18.08	211	170	12	8
Banerjee et al^3^	2009	IV	3	3	3/0	3/0	23	20-25	23	20-25	14	3	3	3	3
Narbona et al^13^	2023	IV	379	8	NR	NR	20	NR	23	17-24	30	NR	NR	8	NR
Fritsch et al^6^	2010	IV	4	4	4/0	4/0	23.75	16-42	23.75	16-42	35.75	3	0	3	0
Augusti et al^1^	2015	IV	12	9	12/0	9/0	28.42	17-61	32.7	19-61	23.11	10	NR	9	NR
Park et al^15^	2015	IV	570	9	NR	9/0	NR	NR	28.8	18-49	27.33	NR	NR	6	2

*NR*, not reported; *PSF*, postage stamp fracture; *M*, male; *F*, female.

Age at surgery refers to the patient's age at the time of their first arthroscopic Bankart repair. Time to fracture indicates the number of months between the initial stabilization and the occurrence of a postage stamp fracture. Collision sports (eg, rugby, football) and noncollision sports (eg, baseball, volleyball, handball, soccer) were categorized separately.

#### Suture anchor characteristics

Anchor quantity in all patients with and without fracture was reported in 5 studies.^1^^,^^3^^,^^6^^,^^12^^,^^14^ The greatest full-sample mean anchor quantity was 5.3 (n = 257).^12^ In that same study, the mean anchor quantity among PSF patients was 5.08 (n = 13). Nakagawa et al found that there was no significant difference in incidence of PSF with regards to anchor quantity; however, recurrence of instability increased with fewer anchors. Nakagawa et al did conclude that postoperative instability was associated with PSF.^12^ In 3 other studies, total patient mean anchor quantity was the same or greater than mean anchor quantity in those with PSF.^3^^,^^6^^,^^14^ No study mentioned a significant relationship between anchor quantity and PSF. There are 5 studies which reported anchor diameter in fractured shoulders, 4 of which had a median anchor diameter of 2.9 mm or higher with a range of 1.4 mm to 5 mm across studies.^1^^,^^3^^,^^6^^,^^12^^,^^15^ Only one study noted anchor diameter across their full sample.^1^ Anchor diameter was not mentioned in any study as having a relationship to incidence of PSF. Of the 4 studies that reported knotted vs. knotless suture use, 3 provided data for both the full sample and fracture patients, 2 of which had more fractures using knotless sutures.^1^^,^^3^^,^^6^^,^^12^ One study showed all fractures occurring with knotted sutures, however they did not report full sample proportions.^12^ In the 4 studies reporting anchor material in patients with and without fracture, 2 used only absorbable anchors (n = 15), 1 used only nonabsorbable (n = 257), and 1 employed both (2 nonabsorbable, 2 absorbable).^1^^,^^3^^,^^6^^,^^12^ One study (n = 379, PSF incidence = 8) concluded that PSF could be associated with bioabsorbable anchors, with 75% of their PSF being in patients who received bioabsorbable anchors.^13^ Another study had 77.7% of their PSF occur in patients with bioabsorbable anchors (n = 570, PSF incidence = 9).^15^ Neither of these studies reported proportion of total patients that received absorbable vs. nonabsorbable anchors. One study (n = 279, PSF incidence = 13) found no significant difference between metal and soft nonabsorble anchors regarding PSF incidence.^12^ Suture anchor characteristics are summarized in Table III.

**Table III:** Technical characteristics.

Author	Sample size	PSF incidence	Median diameter, mm (total)	Median diameter, mm (fracture)	Mean anchor quantity (total)	Mean anchor quantity (fracture)	Knot-tying/knotless (total)	Knot-tying/knotless (fracture)	Absorbable (total)	Absorbable (fracture)	Nonabsorbable (total)				Nonabsorbable (fracture)			
											Polymer or ceramic	Metal	All-suture	PEEK	Polymer or ceramic	Metal	All-suture	PEEK
Park et al^14^	39	19	NR	NR	4	4	NR	NR	NR	NR	NR	NR	NR		NR	NR	NR	NR
Nakagawa et al^12^	257	13	NR	1.4 (1.4-2.4)	5.3	5.08	NR	13/0	0	0	0	128	129	0	0	5	8	0
Banerjee et al^3^	3	3	NR	3.9 (3.9-3.9)	3.33	3.33	0/3	0/3	3	3	0	0	0	0	0	0	0	0
Narbona et al^13^	379	8	NR	NR	NR	NR	NR	NR	NR	6	NR	NR	NR	0	0	2	0	0
Fritsch et al^6^	4	4	NR	3.5 (2-5)	3.5	3.33	0/4	0/4	3	3	0	0	0	1	0	0	0	1
Augusti et al^1^	12	9	3.01∗	3.1 (2.8-3.5)	2.42	2.6	10/2	7/2	12	9	0	0	0	0	0	0	0	0
Park et al^15^	570	9	NR	2.9 (2.9-3.2)	NR	3.22	NR	NR	NR	7	NR	NR	NR	NR	0	2	0	0

*NR*, not reported; *PSF*, postage stamp fracture; *PEEK*, polyether ether ketone.

Summary of the data extracted representing characteristics related to suture anchors and their use.

∗ Only reported mean.

#### Fracture characteristics

All PSF were associated with recurrent instability, with one study mentioning this as a significant relationship.^12^ Three studies reported presence of osteolysis in patients with and without fracture with incidences ranging from 23.5% to 100% of patients.^1^^,^^14^^,^^15^ In 2 of those studies, osteolysis was found in 55.5% (n = 570, PSF incidence = 9) and 100% (n = 12, PSF incidence = 9) of patients.^1^^,^^15^ The presence of osteolysis made up more than 50% of fracture patients in 4 of 5 studies that reported the value in this group.^1^^,^^12^^,^^13^^,^^15^ One of those studies cited this as a significant risk factor for PSF by odds ratio, and another concluded presence of osteolysis may increase the risk.^15^ A summary of osteolysis can be seen in Table IV. Only one study commented on insertion angle, saying insertion angle did not affect glenoid rim fracture incidence.^14^

**Table IV:** Osteolysis.

Author	Sample size	PSF incidence	Osteolysis (total)	Osteolysis (fracture)
Park et al^14^	39	19	10	4
Nakagawa et al^12^	257	13	NR	9
Banerjee et al^3^	3	3	NR	NR
Narbona et al^13^	379	8	NR	8
Fritsch et al^6^	4	4	NR	NR
Augusti et al^1^	12	9	12	9
Park et al^15^	570	9	134	5

*NR*, not reported; *PSF*, postage stamp fracture.

Summary of data extracted related to the presence of osteolysis.

## Discussion

This systematic review analyzes patient-specific and anchor-specific factors and their potential relationship to PSF risk after arthroscopic Bankart repair. A previous review identified male sex, age < 25 years, sport participation, use of ≥ 3 anchors, use of conventional knot-tying anchors, and the presence of osteolysis as risk factors for PSF.^19^ However, that review did not take into account total patient samples regardless of fracture, increasing susceptibility to selection bias.^10^ When taking whole patient samples into consideration, it appears that patients with PSF after arthroscopic Bankart repair were older on average than the whole patient sample and had fewer anchors. Other factors that appear to be related to PSF include participation in athletics—particularly collision sports and high risk sports—presence of osteolysis and use of absorbable anchors. Whether patient gender or anchor type play a role in PSF requires further discussion and research.

### Patient-specific factors

While previous research says PSFs are fairly common, here, the 3 reviewed studies with more than 40 patients (n = 257, 379, and 570) show an incidence of 5%, 2.1%, and 1.58%, respectively.^12^^,^^13^^,^^15^^,^^19^ While male patients make up a majority of the reported PSFs, it remains to be seen whether this can be considered a risk factor. Our findings challenge this assumption by examining gender distribution across the full cohort of patients undergoing arthroscopic Bankart repair, not just among those who sustained fractures. While our review found most reported PSF cases occurred in male patients, this mirrors the overall male predominance in the surgical population; all included study samples were either entirely or predominantly male. In the few studies that included female patients, the incidence of PSF did not differ between genders.^12^^,^^15^ These findings suggest that prior associations between male gender and PSF may reflect sampling bias rather than a true sex-specific predisposition. This distinction is critical for guiding future research and clinical decision-making, as it highlights the importance of contextualizing demographic risk factors within the full operative population rather than among fracture cases alone. Younger age has also been stated as a potential risk factor for PSF; however, as with gender, these conclusions were often based solely on analyses of patients who sustained fractures, without contextualizing age within the broader operative sample.^19^ Interestingly, in the studies that reported both, the average age of patients who experienced PSF was consistently the same or higher than that of the overall sample. This suggests that younger age may not be an independent risk factor for PSF but rather a reflection of the younger demographic commonly undergoing shoulder stabilization surgery. Future research should also attempt to control for activity level when analyzing the relationship between age and postsurgical complication, as this is a possible confounder. This brings us to the next patient-related factor analyzed in this review—participation in sports. Previous research has suggested that athletic participation may increase the risk of PSF.^19^ Our findings reinforce this association while expanding the analysis to include the full cohort of patients undergoing Bankart repair. Unlike age and gender, this association appears to persist even when contextualized within the broader sample, suggesting that the mechanical demands and repetitive stress inherent to certain sports may meaningfully elevate the risk of PSF. However, future research should attempt to delineate whether this relationship persists by controlling for postoperative recurrent instability. Athletic participation is a risk factor for recurrent instability and recurrent instability is a risk factor for PSF.^12^ Since recurrent instability may lie along the causal pathway between athletic participation and PSF, it is a potential effect modifier.

### Technique-specific factors

Technical variables and their possible relationship to PSF were also evaluated in this review. Prior research has implicated greater anchor quantity as a potential risk factor for PSF.^19^ While previous biomechanical data supports this conclusion, our analysis of full cohort data does not.^9^ In fact, most studies reviewed reported similar or even higher average anchor quantities in the overall patient population compared to those who developed PSF. One study included in both the prior and current review found no significant association between anchor number and PSF, while also demonstrating that fewer anchors were linked to higher rates of postoperative instability, itself a proposed contributor to PSF.^12^ Taken together, these findings suggest that suture anchor quantity alone is unlikely to independently contribute to fracture risk. Instead, arthroscopic Bankart repair with suture anchors, independent of anchor count, may predispose the glenoid to fracture because of localized stress concentration, particularly in the context of trauma or recurrent instability. Surgeons may want to prioritize postoperative stability with repair over using fewer anchors, as instability may be a stronger contributor to complications.^12^ Further, biomechanical studies need to be interpreted in context; cadaveric or synthetic models do not account for biological healing and remodeling processes present in live patients. There was not enough data to report whether anchor diameter plays a role in fracture development. The use of knotted vs. knotless sutures in fracture development is ambiguous because of a lack of data and inconsistency in reporting. The 2019 systematic review had reported that Frisch et al used all knotted sutures; however, 3 patients could have been treated with knotless sutures, as they mention the use of polyether ether ketone SutureTaks (Arthrex, Naples, FL, USA) which come knotless.^6^^,^^19^ Further, the 2019 systematic review noted all of Augusti et al's sutures as knotted; however, it appears 2 of their patients were treated with Impact suture anchors, which also come knotless.^1^^,^^19^ Further research needs to be more specific in their reporting of suture anchor type if any relationship is to be delineated with confidence. It appears that the use of absorbable suture anchors could increase the risk of PSF, as a majority of PSF cases occurred in patients who received bioabsorbable anchors. A possible mechanism could involve the disappearance of a reinforcing presence of the anchor. Augusti et al believe that as these anchors degrade, they may leave behind areas of osteolysis or reduced local bone integrity, weakening the glenoid rim and making it more susceptible to fracture.^1^ As osteolysis has been shown in this study to be a possible contributor to PSF risk, techniques that limit the risk of osteolysis, such as using nonabsorbable anchors, may be considered. However, these findings are difficult to interpret without knowing the proportion of all patients who received each anchor type. Without these denominators, any association between anchor material and fracture risk may reflect underlying usage patterns rather than a causal relationship.

#### Limitations

While this review improves the growing body of knowledge on the topic of arthroscopic Bankart repair, acknowledging its limitations is necessary to contextualize its findings. First, PSFs remain a relatively rare complication of Bankart repair, and the overall number of reported cases is low. This limits the statistical power to detect meaningful associations. Second, the total volume of literature on PSF is sparse, and the studies available are almost exclusively retrospective case series or cohort studies, which introduces the potential for selection bias and limits the strength of any causal inferences. Our response to this was to employ a descriptive synthesis approach. While an appropriate data analysis technique, it is limited by lack of quantification, limiting the ability for statistical testing and no assessment of heterogeneity inherent to meta-analysis.

Furthermore, the sample populations across studies were predominantly composed of young men. While this may reflect the typical demographic undergoing arthroscopic Bankart repair with placement of suture anchors, it introduces a potential limitation in the applicability of findings to other patient groups, such as women or older individuals. Whether this homogeneity reflects a true biological predisposition or merely the characteristics of those at risk for shoulder instability remains uncertain.

In addition, because the underlying data are drawn from published case series, there may be a publication bias toward unusual or severe cases of PSF, and the true incidence and spectrum of fracture presentations may be underrepresented. Future research would benefit from larger, prospective registries or multicenter databases that include standardized reporting of both surgical technique and postoperative outcomes for all patients undergoing Bankart repair. Such efforts would enhance the validity and generalizability of future analyses and may help clarify which patient- or technique-specific factors truly contribute to PSF risk.

## Conclusion

PSF is an uncommon but significant complication following arthroscopic Bankart repair. This review provides an updated synthesis of both patient- and technique-specific factors that may influence PSF risk, offering a broader context by considering entire operative cohorts rather than only those who sustained fractures. While prior assumptions linked male gender, younger age, higher anchor quantity, and knotted sutures to increased fracture risk, our findings suggest these relationships are more nuanced. It is possible that a lower quantity of suture anchors and the use of absorbable anchors may play a role in PSF incidence. Age was not an independent risk factor, as PSF patients were of similar or greater age than the overall cohort. Athletic participation and osteolysis remain promising areas for further investigation, particularly in relation to recurrent instability. Ultimately, this review highlights the need for higher-quality, prospective data with standardized reporting to refine our understanding of PSF risk and inform surgical decision-making. Until then, clinicians should interpret existing risk factors cautiously, recognizing the limitations of the current evidence base and the complex interplay between surgical technique, biological remodeling, and patient activity level in determining outcomes.

## Disclaimers:

Funding: No funding was disclosed by the authors.

Conflicts of interest: No author, nor any member of their family, received any financial remuneration related to the subject of this article.
